# Kanzan cherry blossom and its active compounds attenuate methylglyoxal-induced skin fibroblast senescence through RAGE/NF-κB suppression and ECM restoration

**DOI:** 10.3389/fnut.2026.1927058

**Published:** 2026-10-28

**Authors:** Limin Ouyang, Huiji Zhou, Binrui Yang, Xue Zhang, Xuefei Cai, Jiawei Zhang, Bo Li, Liang Chen

**Affiliations:** Healthy Aging Research Center, Nutrilite Health Institute, Amway (Shanghai) Innovation & Science Co., Ltd., Shanghai, China

**Keywords:** anti-glycation, *Cerasus serrulata* ‘Sekiyama', extracellular matrix, flavonoids, phenolic acids, RAGE/NF-κB, skin aging

## Abstract

**Introduction:**

The accumulation of advanced glycation end products (AGEs) is a key driver of skin aging, accelerating the process through oxidative stress, inflammation, and disruption of extracellular matrix (ECM) homeostasis. *Cerasus serrulata* 'Sekiyama' Kanzan cherry blossom (KCB) is rich in bioactive compounds with antioxidant and anti-glycation potential. However, its compositional variations across flowering stages and their functional relevance remain unclear.

**Methods:**

This study systematically characterized the chemical profile of KCB using UPLC-QTOF-MS and monitored six major active components across four flowering stages using UPLC-MS/MS. The anti-glycation, antioxidant, tyrosinase, and collagenase activities of the identified compounds were evaluated *in vitro*. The protective effects of KCB extract (KCBE) and major active compounds were further investigated in a methylglyoxal (MGO)-induced human skin fibroblast senescence model.

**Results:**

Thirteen compounds were identified, including one organic acid, five flavonoid glycosides, and seven phenolic acids. The half-bloom stage (KCB3) contained the highest levels of the six quantified compounds. 1-*O*-caffeoyl glucoside (CG) was the predominant phenolic acid (8.025 ± 0.203%), while quercetin 3-*O*-6^′′^-*O*-malonyl-β-glucopyranoside (QMG) was the major flavonoid (1.155 ± 0.035%). QMG showed strong anti-glycation activity (IC50 = 0.41 ± 0.01 mM), whereas CG exhibited strong tyrosinase (IC_50_ = 0.19 ± 0.03 mM) and collagenase inhibition (IC_50_ = 2.51 ± 0.90 mM). KCBE, QMG, and CG significantly alleviated MGO-induced cellular senescence, reduced intracellular ROS, restored F-actin organization, and promoted type I collagen deposition. These effects were associated with suppression of RAGE/NF-κB signaling, normalization of the Nrf2/HO-1-mediated antioxidant response, and restoration of ECM-related gene expression.

**Discussion:**

Half-bloom KCB exhibits prominent anti-glycation and anti-skin-aging activities, with CG and QMG representing major bioactive constituents. These findings indicate that KCB and its characteristic phenolic acid and flavonoid constituents may have potential as natural functional ingredients for skin health.

## Graphical Abstract

**Figure:**
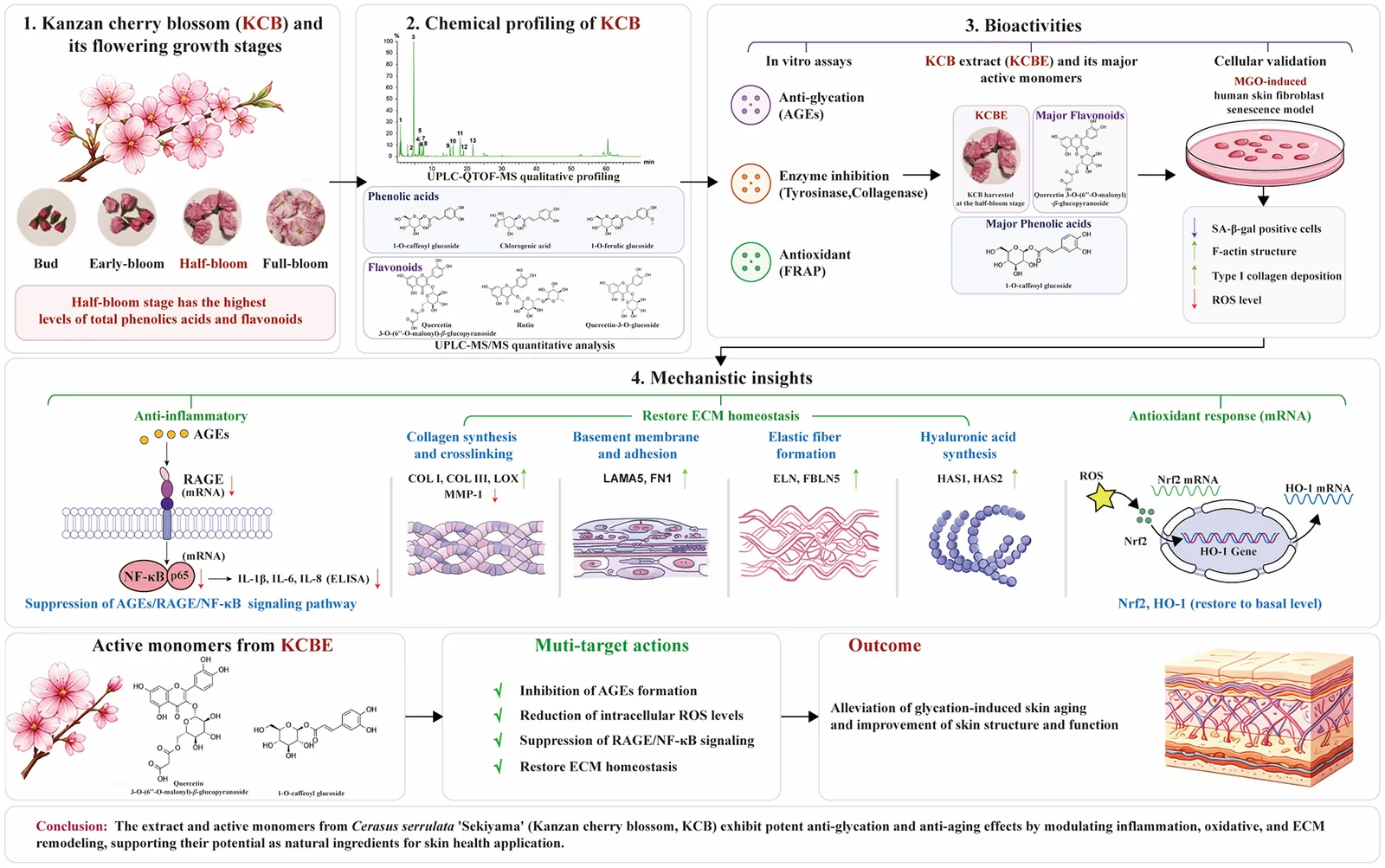

## 1 Introduction

Skin is the largest organ of the human body and serves as a physical barrier protecting against external environmental stressors. It is primarily composed of the epidermis and dermis (1). The epidermis, the outermost layer, consists mainly of keratinocytes (2), while the dermis is enriched with extracellular matrix (ECM) components secreted by human skin fibroblasts (Fbs), including collagen, elastin, and hyaluronic acid. Together, these components maintain the mechanical strength, elasticity, and hydration of the skin (3, 4). The ECM serves as a fundamental structural and functional scaffold for cells, playing essential roles in maintaining tissue integrity, regulating inflammatory responses, and modulating cellular function (5). Continuous ECM remodeling and its dysregulation are recognized as key hallmarks of cellular senescence and have been included among the recently proposed hallmarks of aging (6).

Glycation is considered one of the major mechanisms driving skin aging (7). With aging and metabolic stress, the highly reactive carbonyl compound methylglyoxal (MGO) progressively accumulates *in vivo*, promoting non-enzymatic glycation reactions and facilitating the formation of advanced glycation end products (AGEs) (8). Excessive AGEs accumulation has been implicated in multiple age-related diseases, including cancer, cardiovascular disorders, diabetic complications, and neurodegenerative diseases (9, 10). In the skin, AGEs are prone to deposition, leading to structural damage and thereby serving as an important contributor to skin aging (11, 12). In the dermal microenvironment, accumulated AGEs interact with the receptor for advanced glycation end products (RAGE) on Fbs, triggering excessive reactive oxygen species (ROS) production and activating inflammatory signaling pathways such as NF-κB (9). This promotes Fbs senescence, characterized by increased Senescence-associated β-galactosidase (SA-β-gal) activity (13). Senescent Fbs further exacerbate oxidative stress and secrete matrix metalloproteinases (MMPs) and pro-inflammatory cytokines (14), resulting in collagen degradation and ECM disruption, ultimately accelerating skin aging. Therefore, the identification of natural, safe, and effective plant-derived compounds capable of inhibiting glycation-associated aging has become a research focus in the development of anti-aging products.

*Cerasus serrulata* ‘Sekiyama', commonly known as Kanzan cherry blossom (KCB), a representative double-flowered late-blooming cherry cultivar of the Rosaceae family, is widely cultivated in East Asia. It exhibits high ornamental and edible value due to its vibrant flowers and simultaneous leaf and blossom development. This species contains bioactive constituents including flavonoids, polyphenols, organic acids, volatile oils, and pigment-related components (isosakuranetin) (15). These compounds possess strong antioxidant activity and free radical scavenging capacity, thereby alleviating oxidative stress-induced skin damage and demonstrating promising potential in skin anti-aging applications (16). Previous studies have shown that KCB extract (KCBE) can inhibit AGE formation *in vitro* and alleviate glyoxal-induced Fbs apoptosis (17). In Japan, this plant has a long history of consumption, being widely used in traditional foods such as cherry blossom rice cakes and bread (18). In 2022, it was officially approved as a new food raw material by the National Health Commission of China, providing regulatory support for its development from an ornamental plant into functional food and health-related products (16). However, despite increasing attention in recent years, a systematic understanding of the bioactive constituents in the KCB and their underlying molecular mechanisms remains limited, which severely restricts further high-value industrial application of this botanical resource.

In this study, thirteen chemical compounds were identified from KCB samples. For the first time, the dynamic changes of six key active compounds were investigated across four distinct flowering growth stages, namely the flower bud stage (KCB1), early-bloom stage (KCB2), half-bloom stage (KCB3), and full-bloom stage (KCB4). This analysis identified KCB3 as the flowering stage with the highest cumulative content of the six target compounds. Based on the results of content quantification and four enzymatic assays, 1-*O*-caffeoyl glucoside (CG) and quercetin 3-*O*-6^′′^-*O*-malonyl)-β-glucopyranoside (QMG) were preliminarily judged as the major active monomers in KCB3. An MGO-induced Fbs glycation senescence model was then established to investigate the effects of the extract and its two key compounds on senescence phenotypes, oxidative stress, and inflammatory microenvironments. Furthermore, their regulatory effects on ECM-related genes involved in collagen synthesis and degradation, basement membrane stability, elasticity, and cell adhesion were analyzed, providing a scientific foundation for the development of KCB3 as a functional anti-aging ingredient.

## 2 Materials and methods

### 2.1 Instruments

Milli-Q ultrapure water system (Jizhou Chemical Technology Co., Ltd., Shanghai, China); WF-600EHT ultrasonic cleaner (Wufang Ultrasonic Equipment Co., Ltd., Ningbo, China); MS3002S electronic analytical balance; Waters SYNAPT XS Ultra Performance Liquid Chromatography–Quadrupole Time-of-Flight Mass Spectrometer (Waters Corporation, Milford, MA, USA); mini vortex mixer (Shanghai Titan Technology Co., Ltd., Shanghai, China); DualRange analytical balance XS205DU (Mettler Toledo International Trading Co., Ltd., Shanghai, China); SK8210LHC ultrasonic cleaner (Kudos Ultrasonic Instrument Co., Ltd., Shanghai, China); FlexStation3 multi-mode microplate reader (Molecular Devices, San Jose, CA, USA); 7,300 Plus Real-Time PCR System (Applied Biosystems, Foster City, CA, USA); ECLIPSE Ti Microscope (Nikon Corporation, Tokyo, Japan); Varioskan LUX Multifunctional Microplate Reader and HERAcell 150i Cell Incubator (Thermo Fisher Scientific, Waltham, MA, USA); Liangfang Cell Expansion System (Regenovo Biotechnology Co., Ltd., Hangzhou, China).

### 2.2 Materials and reagents

KCB were collected from Xibuqian Village, Culai Town, Daiyue District, Taian City, Shandong Province, China and were authenticated by Dr. Xuefei Cai of Amway Botanical Research Center, China (Supplementary Figure 1). The flowers are pink, brightly colored, and free from disease lesions, with numerous and dense petals. Flowering occurs in early to mid-April. The flowers were dried at 35–60 °C for 11–12 h, and the dried materials were then stored at 0–5 °C.

Acetonitrile and methanol (HPLC grade, Fisher Chemical, Waltham, MA, USA); ethanol and formic acid (analytical grade, Shand Standard Technology Co., Ltd., Shanghai, China); 1-*O*-caffeoyl glucoside, 1-*O*-ferulic glucoside, quercetin 3-*O*-(6^′′^-*O*-malonyl)-β-glucopyranoside, rutin, chlorogenic acid, quercetin-3-*O*-glucoside (Pusi Biotechnology Co., Ltd., Chengdu, China); bovine serum albumin (BSA) lyophilized powder, 6-hydroxyl-2,5,7,8-tetra-methylchroman-2-carboxylic acid (Trolox), tyrosinase, collagenase from *Clostridium histolyticum* (ChC) (≥125 CDU/mg solid), N-[3-(2-furyl)acryloyl]-Leu-Gly-Pro-Ala (FALGPA), dimethyl sulfoxide (DMSO), aminoguanidine hydrochloride (AG), methylglyoxal and Triton X-100 (Sigma-Aldrich, St. Louis, MO, USA); Cell Counting Kit-8 (CCK-8) (SunView Technology Co., Ltd., Shenzhen, China); Total Antioxidant Capacity Assay Kit, Ferric Reducing Ability of Plasma (FRAP) Kit and Cell Senescence β-Galactosidase Staining Kit (Beyotime Biotechnology Inc., Shanghai, China); Human IL-1β ELISA Kit, Human IL-6 ELISA Kit and Human IL-8 ELISA Kit (Boster Biological Technology, Wuhan, China); Human skin-derived fibroblasts (Regenovo Biotechnology Co., Ltd., Hangzhou, China); DMEM high-glucose medium (Gibco, Thermo Fisher Scientific, Waltham, MA, USA); premium-grade fetal bovine serum (ExCell Biotechnology Co., Ltd., Suzhou, China);2′7′-Dichlorodihydrofluorescein diacetate (H2DCFDA) and FPS-ZM1 (MedChemExpress LLC, Monmouth Junction, NJ, USA); Anti-Collagen I antibody [EPR7785] and Goat Anti-Rabbit IgG H&L (Alexa Fluor^®^ 488) (Abcam Ltd, Cambridge, UK); phalloidin (AAT Bioquest, Pleasanton, CA, USA); DAPI and TRIzol (Invitrogen, Carlsbad, CA, USA); PrimeScriptTM 1st Strand cDNA Synthesis Kit and TB Green Premix Ex TaqTM (Takara Biomedical Technology (Beijing) Co., Ltd., Beijing, China). Arbutin and epigallocatechin gallate (EGCG) (Shanghai Titan Technology Co., Ltd., Shanghai, China); Ultrapure water was provided by a Milli-Q laboratory water purification system (Merck Millipore, Billerica, MA, USA). All chemicals were of analytical or reagent grade and used without further purification.

### 2.3 Methods

#### 2.3.1 UPLC-QTOF-MS identification of chemical constituents

Powdered cherry blossom (1.01 g) was mixed with 50 ml of 70% (v/v) ethanol. The mixture was heated for 10 min and then ultrasonicated at 51 kHz for 30 min. The solution was filtered through a 0.22 μm membrane filter, and the filtrate was directly used for UPLC-QTOF-MS analysis.

UPLC conditions: ACQUITY UPLC Premier HSS T3 column (100 × 2.1 mm, 1.8 μm); mobile phase A: 0.1% formic acid aqueous solution, mobile phase B: acetonitrile; gradient elution; flow rate: 0.3 ml·min^−1^; detection wavelengths: 254 nm, 280 nm, 360 nm; injection volume: 2 μl. Gradient program: 0–3 min, 95% A; 3–23 min, 95%−78% A; 23–24 min, 78%−73% A; 24–35 min, 73%−65% A; 35–37 min, 65%−20% A; 37–39 min, 20% A; 39–40 min, 20%−95% A.

MS conditions: Ion source: ESI, negative ion mode. First-order MS: scan range 50–1,700 *m/z*; nebulizer gas: 50 psi; curtain gas: 35 psi; ion source voltage: 5,000 V; ion source temperature: 500 °C; declustering potential (DP): 100 V; collision energy (CE): 10 eV. Second-order MS: scan range 50–1,250 *m/z*; DP: 100 V; CE: 40 eV; collision energy spread: 20 eV. Data were acquired using Waters MassLynx (version 4.2) and processed with Waters Connection HUB (version 3.2). Compounds were preliminarily screened by matching mass spectral data with the Waters UNIFI (version 3.2) database, then confirmed by retention time, UV maximum absorption, accurate mass, molecular formula, comparison with reference standards, and literature-reported fragmentation rules.

#### 2.3.2 UPLC-MS/MS quantification of constituents

KCB samples collected at four different flowering stages, designated as KCB1, KCB2, KCB3, and KCB4, were individually ground into a fine powder. From each stage, 1.02 g of the powder was accurately weighed and mixed with 50 ml of 70% (v/v) ethanol. The mixture was heated for 10 min and then ultrasonicated at 51 kHz for 30 min. The resulting solution was directly injected for analysis after passing through a 0.22 μm membrane filter.

Triple quadrupole MS conditions were referred to previous literature with minor change (19). Ion source: ESI; ionization mode: negative; detection mode: multiple reaction monitoring (MRM). Spray voltage: 2.5 kV; desolvation temperature: 500 °C; desolvation gas flow: 1,000 L·Hr^−1^. Data were acquired with Xcalibur 4.1 software (Waters) and exported as Excel files. Mass parameters for target compounds are listed in Table 1. The solution (0.2 mg/ml) was filtered through a 0.22 μm membrane, and the filtrate was diluted 100 times before injection and analysis.

**Table 1:** Mass spectrometry parameters of chemical components.

Compounds	Mode	Quantitative ion pair (CE/eV)	Qualitative ion pair (CE/eV)
1-*O*-caffeoyl glucoside	[M–H]^−^	341.063>160.961 (18)	341.063>132.99 (40)
1–*O*–ferulic glucoside	[M–H]^−^	355.079>174.963 (18)	355.079>159.935 (36)
Quercetin−3–*O*–glucoside	[M–H]^−^	463.127>300.188 (26)	463.127>270.995 (44)
Quercetin 3–*O*–(6^′′^–malonyl)–β–glucopyranoside	[M–H]^−^	549.064>300.122 (32)	549.064>270.994 (50)
Rutin	[M–H]^−^	609.122>300.12 (38)	609.122>270.991 (58)
Chlorogenic acid	[M–H]^−^	353.063>190.964 (16)	353.063>127.401 (34)

#### 2.3.3 Extract preparation

Dried KCB were ground and sieved through a 20-mesh sieve. The powder (50.01g) was extracted twice with 12 volumes of water under reflux for 1 h each time. The extracts were combined, cooled to room temperature, and vacuum-filtered through a 0.45 μm membrane. The clear filtrate was concentrated under reduced pressure using a rotary evaporator to obtain a small-volume concentrated extract. The aqueous concentrate was freeze-dried and finally achieved 7.81g KCBE. The yield of the aqueous extract was 15.62%. KCBE was stored at 4 °C until use.

#### 2.3.4 Enzymatic and antioxidant assays

The antiglycation capacity, anti-tyrosinase activity, and collagenase inhibition activity of the samples were analyzed in 96-well microplates according to a previously described protocol (20).

FRAP total antioxidant capacity, 0.15, 0.3, 0.6, 0.9, 1.2, and 1.5 mM FeSO_4_ solutions were used for the calibration curve, with the FRAP value being represented by a FeSO_4_ concentration. The regression equation of the curve was used to calculate equivalent concentration 1 (EC_1_) for each sample. EC_1_ is defined as the concentration of antioxidant giving an absorbance equivalent to that of 1.0 mM FeSO_4_ determined using its own calibration curve (20).

#### 2.3.5 Cell culture, induction and treatment

Human skin Fbs were cultured in Dulbecco's Modified Eagle Medium (DMEM) supplemented with 10% fetal bovine serum (FBS) and 1 × penicillin/streptomycin. Cells were maintained at 37 °C in a humidified incubator with 5% CO_2_ (~95% humidity). The culture medium was replaced every 2–3 days. Cells were passaged upon reaching approximately 80% confluence and maintained under standard culture conditions. For subsequent experiments, cells were seeded at appropriate densities into 96-well plates for cell viability assays, 24-well plates for morphological and fluorescence staining, and 6-well plates for ELISA and qPCR analyses, prior to specific treatments. For senescence induction, cells were treated with MGO. Experimental groups were supplemented with 1 mM aminoguanidine hydrochloride (AG) or 5 μm FPS-ZM1 as positive controls, and treated with KCBE, QMG or CG as experimental interventions. Cells were cultured for 3 days with half-medium replacement daily.

#### 2.3.6 Cell viability assay

Cell viability was assessed using a CCK-8 assay. Fbs were seeded at a density of 1 × 104 cells per well and incubated overnight under standard conditions (37 °C, 5% CO_2_, 95% humidity).

Cells were treated with varying concentrations of KCBE (100–10,000 μg/ml), QMG (1–200 μm), or CG (1–200 μm). Wells containing serum-free DMEM served as controls. After 48 h of treatment, 100 μl of CCK-8 solution was added to each well and incubated for 2 h at 37 °C. Absorbance was measured at 450 nm using a microplate reader.

#### 2.3.7 SA-β-gal staining

For SA-β-gal staining, cells were processed using a commercial kit according to the manufacturer's instructions to evaluate cellular senescence. Cells were incubated overnight at 37 °C (1 mM AG was used as a positive control). Images were acquired using an inverted microscope (20×). For each well, multiple random fields were captured to count the number of SA-β-gal-positive cells and total cells, and representative images were recorded. The positive rate and its relative changes were subsequently calculated.

#### 2.3.8 Fluorescent staining of F-Actin

The cytoskeletal structure of filamentous actin (F-actin) in Fbs was evaluated by fluorescent staining. After treatments (1 mM AG was used as a positive control), cells were fixed with 4% paraformaldehyde for 15 min, followed by permeabilization with 0.1% Triton X-100 and washing with PBS (3 times).

Cells were incubated with phalloidin at room temperature in the dark for 30 min. Nuclei were counterstained with DAPI. After washing, images were captured using a fluorescence microscope (20×) to evaluate cytoskeletal morphology, and representative images were recorded.

#### 2.3.9 Immunofluorescence staining of COL I deposition

Type I collagen expression was evaluated by immunofluorescence staining. After treatments (1 mM AG was used as a positive control), cells were fixed with 4% paraformaldehyde for 15 min without permeabilization. Cells were washed with PBS and blocked with 2% BSA at room temperature. Cells were incubated with anti-COL I primary antibody at 4 °C overnight, followed by incubation with a fluorescent secondary antibody. Nuclei were stained with DAPI. After washing, images were captured directly in the plates using a fluorescence microscope (20×). ImageJ software was used to quantify the mean fluorescence intensity to evaluate relative changes among groups.

#### 2.3.10 Intracellular ROS detection

Intracellular ROS levels in Fbs were measured using H2DCFDA staining. After treatments (1 mM AG was used as a positive control), cells were washed with PBS and incubated with H2DCFDA in the dark for 35 min at 37 °C. After incubation, the dye was removed, and cells were washed with PBS to eliminate background fluorescence. Fluorescent images were captured directly in the wells using a fluorescence microscope (20×). ImageJ software was used to quantify the mean fluorescence intensity to evaluate relative changes among groups.

#### 2.3.11 Measurement of inflammatory cytokines (ELISA)

After designated treatments (5 μM FPS-ZM1 was used as a positive control), cell culture supernatants were collected and centrifuged to remove cellular debris. Levels of IL-1β, IL-6, and IL-8 were measured using ELISA kits according to the manufacturer's instructions. Absorbance at 450 nm was recorded using a microplate reader.

#### 2.3.12 mRNA extraction and quantitative real-time PCR (qPCR)

Total RNA was extracted from cells using TRIzol reagent after treatments (5 μM FPS-ZM1 was used as a positive control). First-strand cDNA was synthesized using a 1st Strand cDNA Synthesis Kit according to the manufacturer's protocol. qPCR was performed using a SYBR Green master mix on a real-time PCR system to assess ECM- and senescence-related gene expression, with specific primer sequences detailed in Supplementary Table 1. Relative gene expression levels were calculated using the 2^(−ΔΔ*Ct*)^ method, with GAPDH serving as the internal reference control.

#### 2.3.13 Statistical analyses

Data were analyzed and plotted using Excel and GraphPad Prism 9.0 software. All experiments were performed in three parallel replicates, and the results were expressed as mean ± standard deviation (SD), and significance was evaluated by one-way analysis of variance (ANOVA) and Dunnett's test.

## 3 Results

### 3.1 UPLC-QTOF-MS identification of chemical constituents in KCB

The KCB was analyzed by UPLC-QTOF-MS (Figure 1). Thirteen compounds were tentatively identified based on quasi-molecular ions, MS/MS fragments, reference standards, and literature data (Table 2). As a Prunus species (Rosaceae), KCB petals are rich in polyphenols, including flavonoid glycosides and caffeoyl derivatives (17, 19, 21).

**Figure 1:**
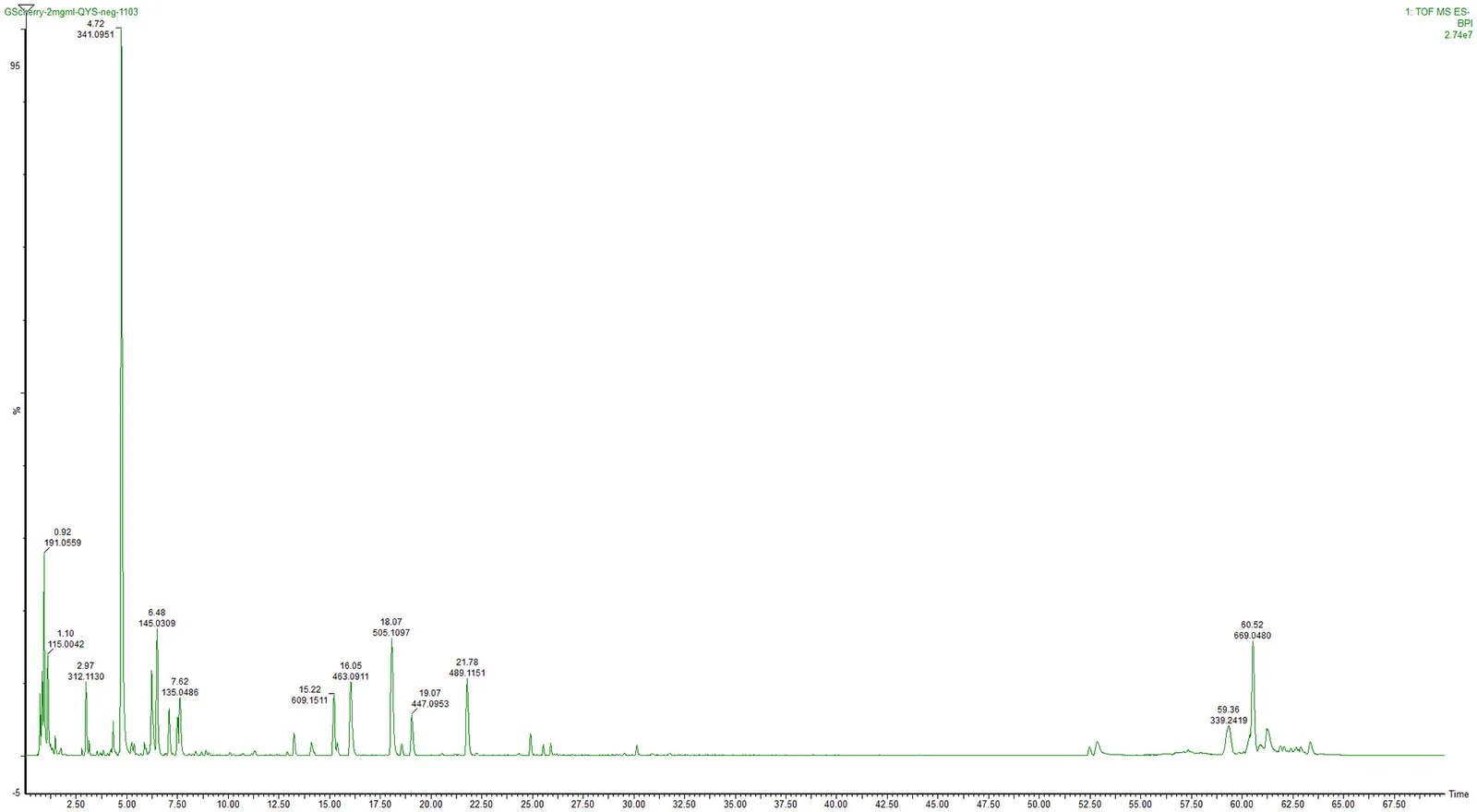
Base peak chromatogram-negative ion mode of KCB.

**Table 2:** Identification results of main components in KCB.

No.	T (min)	Ion	*m/z*	MS/MS data	Molecular weight	Name	Formula	Identification method
1	0.92	[M–H]^−^	191.0559	175.0278; 161.0486; 131.0488; 85.0301	192.12	Citric acid	C6H8O7	Reference
2	4.32	[M–H]^−^	353.0907	341.0950; 191.0559; 179.0383; 130.9682	354.31	Neochlorogenic acid	C_16_H_18_O_9_	Reference
3	4.72	[M–H]^−^	341.0950	179.0383; 161.0237	342.30	1–*O*–caffeoyl glucoside	C_15_H_18_O_9_	Standards
4	6.24	[M–H]^−^	353.0907	191.0613; 179.0383; 85.0301	354.31	Chlorogenic acid	C_16_H_18_O_9_	Standards
5	6.48	[M–H]^−^	325.0990	187.0405; 163.0392; 145.0309	326.30	1–*O*–coumaryl–β–*D*– glucoside	C_15_H_18_O_8_	Reference
6	7.08	[M–H]^−^	325.0990	187.0405; 163.0392; 145.0309	326.30	Coumaryl–β–*D*– glucoside isomer	C_15_H_18_O_8_	Reference
7	7.46	[M–H]^−^	355.1064	217.0503; 193.0551; 175.0434	356.33	1–*O*–ferulic glucoside	C_16_H_20_O_9_	Standards
8	7.62	[M–H]^−^	355.1064	262.9871; 179.0383; 135.0486	356.32	6–*O*–ferulic glucoside	C_16_H_20_O_9_	Reference
9	15.26	[M–H]^−^	609.1511	301.0039; 206.9753; 174.9554; 130.9682	610.52	Rutin	C_27_H_30_O_16_	Standards
10	16.05	[M–H]^−^	463.0910	301.0378; 174.9554; 130.9682	464.38	Quercetin −3–*O*– glucoside	C_21_H_20_O_12_	Standards
11	18.07	[M–H]^−^	549.0598	505.0708; 463.0698; 301.0177	550.43	Quercetin 3–*O*–(6^′′^–malonyl)–β–glucopyranoside	C_24_H_22_O_15_	Standards
12	19.05	[M–H]^−^	447.1035	285.0443; 206.9753; 174.9554; 130.9682	448.38	Kaempferol −3–*O*–β–D– glucoside	C_21_H_20_O_11_	Reference
13	21.79	[M–H]^−^	489.1151	429.0858; 285.0443; 206.9753; 174.9606	490.41	Kaempferol −3–*O*–(6^′′^–malonyl)–β–glucopyranoside	C_23_H_22_O_12_	Reference

Compound **3** showed a quasi-molecular ion [M–H]^−^ at *m/z* 341.0950, with the elemental composition C15H18O9. Under collision-induced dissociation, the ester bond cleaved to produce characteristic fragments at *m/z* 179.0383 (caffeic acid) and 161.0237 (hexose residue), diagnostic for caffeoyl compounds (21–23). Combined with reference standard and literature (23), compound **3** was identified as CG. Compound **11** exhibited [M–H]^−^ at *m/z* 549.0598 (C_2_4H_22_O15). Its MS/MS fragments at *m/z* 505.0708, 463.0698, and 301.0177 indicated sequential loss of CO_2_, malonyl group, and hexose, finally yielding the quercetin aglycone ion. Compound **11** was identified as QMG. Malonylated flavonoid glycosides exhibit a stable characteristic fragmentation pattern. First, the terminal carboxyl group of the malonyl group undergoes decarboxylation, producing the fragment ion [M–H−44]^−^ (*m/z* 505). Then, the entire malonyl structural unit can be completely eliminated, yielding the characteristic fragment ion [M–H−86]^−^ (*m/z* 463). The above two neutral loss fragments can serve as specific diagnostic ions for malonylated flavonoid glycosides, which can effectively distinguish acylated from non-acylated flavonoid glycoside constituents. After complete removal of the malonyl group, the intermediate ion at *m/z* 463 (quercetin-3-*O*-glucoside) further undergoes cleavage of the *O*-glycosidic bond, losing a glucosyl residue of 162 Da, and finally forms the characteristic aglycone ion of quercetin at *m/z* 301.0177. Other compounds were identified by retention time, fragmentation patterns, literature, and reference standards (Table 2).

### 3.2 UPLC-MS/MS quantification of KCB at different flowering stages

Based on the qualitative profiling results in Section 3.1, six representative compounds—comprising three phenolic acids and three flavonoids with high peak intensities—were systematically selected for subsequent quantitative monitoring across different flowering stages. Retention times, regression equations and content determination results of six target compounds in KCB at different flowering stages were summarized in Table 3. The types of detected compounds were consistent in KCB from different flowering periods (Figure 2A), while the contents of each compound presented significant differences. The total content of the six compounds ranged from 6.825% to 10.076%. Total content in descending order was: KCB3 (10.076%) > KCB4 (9.471%) > KCB2 (8.902%) > KCB1 (6.825%) (Figure 2B). Among them, the total content of the six compounds was highest at the KCB3. Meanwhile, CG, the major bioactive compound, also showed its maximum content of 8.025 ± 0.203% in this stage. At KCB4, the content of this compound ranked second at 7.718 ± 0.279%, which was still higher than those at the KCB1 and KCB2. In terms of harvesting period, the cumulative content of the six target compounds in KCB3 was the highest among the four flowering stages. The average content of each component ranked in descending order as follows: CG > QMG > chlorogenic acid > quercetin-3-*O*-glucoside > rutin > 1-*O*-ferulic glucoside. Among them, CG and QMG presented the highest contents among all growth stages. The content of CG ranged from 4.164% to 8.025%, while that of QMG varied from 0.943% to 1.491%. They were identified as the major phenolic acid and flavonoid constituents in KCB, respectively. Considering the potential application of KCB products and the feasibility of their subsequent production and practical application, KCB was subjected to aqueous extraction to prepare KCBE. The 13 compounds identified in KCB were also detected in KCBE, indicating that these representative chemical constituents were retained during the aqueous extraction process. The resulting KCBE was subsequently used in the functional evaluation experiments to investigate its biological effects.

**Table 3:** Determination results of the main compounds in the alcohol extract of different flowering degrees of KCB.

Compounds	Regression equation	Linear range (ng·ml^−1^)	Correlation (R^2^)	KCB1	KCB2	KCB3	KCB4
1–*O*–caffeoyl glucoside	*Y*=8,415.7*X –*82,866.5	100–1,600	0.9972	4.164 ± 0.010%	6.619 ± 0.105%	8.025 ± 0.203%	7.718 ± 0.279%
1–*O*–ferulic glucoside	*Y*=7,837.17*X –*15,071.8	10–200	0.9994	0.112 ± 0.003%	0.112 ± 0.004%	0.106 ± 0.002%	0.097 ± 0.003%
Quercetin−3–*O*–glucoside	*Y*=4,933.88*X –*10,623	10–200	0.9979	0.325 ± 0.012%	0.275 ± 0.005%	0.236 ± 0.004%	0.202 ± 0.005%
Quercetin 3–*O*–(6^′′^–malonyl)–β–glucopyranoside	*Y*=1,647.08*X –*3,094.25	10–200	0.9954	1.491 ± 0.040%	1.286 ± 0.033%	1.155 ± 0.035%	0.943 ± 0.016%
Rutin	*Y*=3,903.74*X –*12,946.5	10–200	0.9982	0.206 ± 0.002%	0.169 ± 0.000%	0.147 ± 0.002%	0.126 ± 0.004%
Chlorogenic acid	*Y*=10,322.4*X –*532,994	100–1,600	0.9972	0.527 ± 0.028%	0.442 ± 0.008%	0.408 ± 0.004%	0.386 ± 0.001%
Total amount of compounds				6.825 ± 0.078%	8.902 ± 0.148%	10.076 ± 0.248%	9.471 ± 0.293%

Data are expressed as the mean ± SD (n = 3).

**Figure 2:**
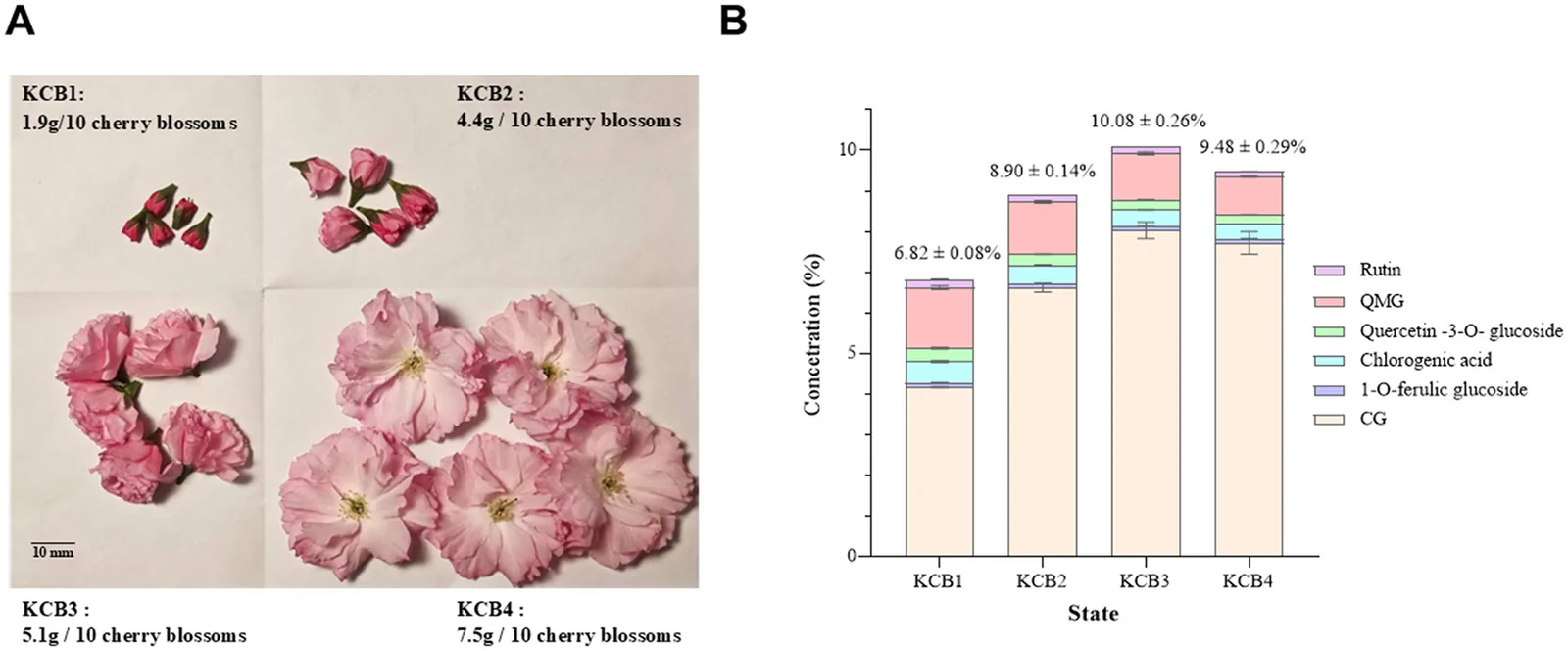
Four flowering periods of KCB. **(A)** Samples of KCB at different flowering stages, where KCB 1–4 correspond to growth stages KCB1–KCB4, respectively. **(B)** The total content of 6 target compounds in different flowering stages of KCB.

### 3.3 Enzyme inhibition and antioxidant effect by KCBE and principal compounds

To further identify the major bioactive constituents of KCBE, six representative compounds were selected for preliminary bioactivity screening. The inhibitory effect on AGEs (Figure 3A) demonstrated that KCBE showed weak anti-glycation activity with an inhibition rate of only 16.13 ± 2.43% at 10 mg/ml. Flavonoids exhibited remarkable AGEs inhibitory capacity in a concentration-dependent manner, while phenolic acids barely exerted anti-glycation effects. The anti-glycation potency of flavonoids decreased in the order: rutin (IC_50_: 0.20 ± 0.01 mM), quercetin-3-*O*-glucoside (IC_50_: 0.37 ± 0.00 mM), QMG (IC_50_: 0.41 ± 0.01 mM). Notably, rutin is a well-documented natural compound with potent anti-glycation property, which is consistent with previous literature reports.

**Figure 3:**
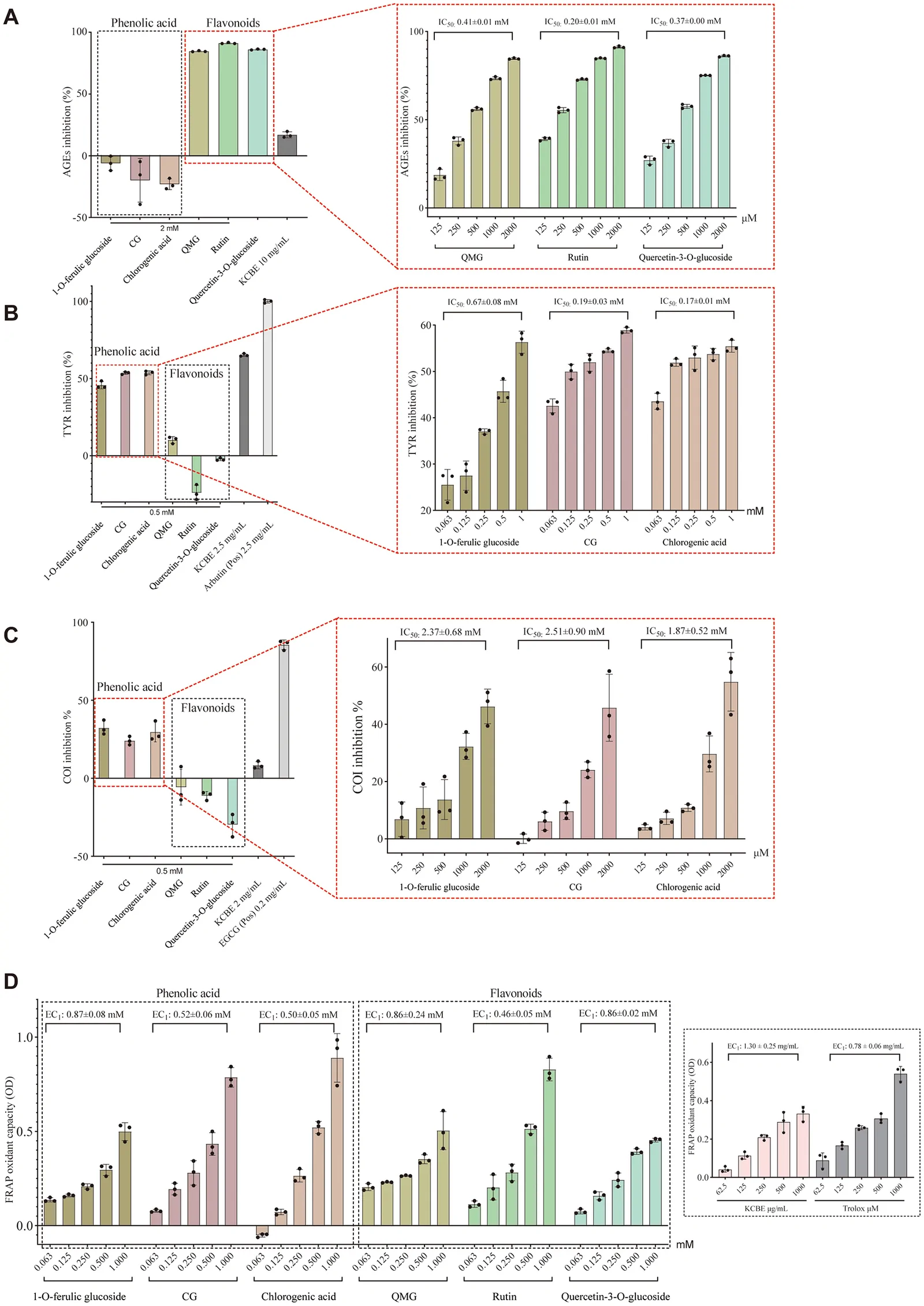
The *in vitro* experimental effects of KCB and its main compounds. **(A)** The inhibitory effect of cherry blossom extract and its main compounds on AGEs. **(B)** The inhibitory effect of cherry blossom extract and its main compounds on tyrosinase. **(C)** The inhibitory effect of cherry blossom extract and its main compounds on Collagenase. **(D)** The antioxidant effect of compounds. Data are expressed as the mean ± SD (*n* = 3).

Tyrosinase inhibitory assay (Figure 3B) indicated that KCBE possessed whitening potential with an inhibition rate of 65.13 ± 0.89% at 2.5 mg/ml, whereas arbutin as the positive control displayed an inhibitory rate of 100.03 ± 1.13% at the same concentration. Phenolic acids were identified as the predominant contributors to tyrosinase inhibition, while flavonoids showed almost no inhibitory activity. All phenolic acids exerted tyrosinase suppression in a concentration-dependent manner, with IC_50_ values of 0.67 ± 0.08 mM, 0.19 ± 0.03 mM, and 0.17 ± 0.01 mM for 1-*O*-ferulic glucoside, CG, and chlorogenic acid, respectively. Both chlorogenic acid and CG possessed significantly stronger tyrosinase inhibitory ability than 1-*O*-ferulic glucoside, demonstrating that the caffeoyl group conferred higher anti-tyrosinase activity than the ferulic group.

Collagenase plays a critical role in degrading dermal collagen and impairing skin structural firmness. KCBE exhibited weak collagenase inhibitory activity (Figure 3C), with merely 8.41 ± 2.28% inhibition at 2 mg/ml; by contrast, the positive control EGCG reached 85.55 ± 3.12% inhibition at 0.2 mg/ml. Among all isolated compounds, only phenolic acids showed prominent anti-collagenase effects, with IC_50_ values of 2.37 ± 0.68 mM, 2.51 ± 0.90 mM, and 1.87 ± 0.52 mM for 1-*O*-ferulic glucoside, CG, and chlorogenic acid, respectively.

FRAP assay was applied to evaluate the antioxidant capacity of KCBE and its main characteristic compounds (Figure 3D). KCBE exhibited moderate antioxidant activity at 1 mg/ml, with a FRAP value equivalent to 0.70 mM Fe^+2^, while the positive control Trolox at 1 mM showed a much higher antioxidant level of 3.36 mM Fe^+2^. All tested compounds presented a concentration-dependent antioxidant effect, and the overall antioxidant potency ranked as follows: rutin (EC_1_: 0.46 ± 0.05 mM), chlorogenic acid (EC_1_: 0.50 ± 0.05 mM), CG (EC_1_: 0.52 ± 0.06 mM), 1-*O*-ferulic glucoside (EC_1_: 0.87 ± 0.08 mM), QMG (EC_1_: 0.86 ± 0.24 mM), quercetin-3-*O*-glucoside (EC_1_: 0.86 ± 0.02 mM).

Overall, phenolic acids mainly contributed to antioxidant, tyrosinase and collagenase inhibition, supporting whitening and anti-aging effects. Flavonoids, especially rutin, dominated antioxidant and anti-glycation activities. The multifunctional bioactivities of KCBE were derived from the synergistic effect of phenolic acid and flavonoid constituents.

### 3.4 Protective effects of KCBE, QMG, and CG against MGO-induced Fbs senescence

Based on the preliminary screening results, QMG and CG were selected as representative active constituents for subsequent cellular validation. To determine the safe concentration range of KCBE, QMG and CG, cell viability was first evaluated using the CCK-8 assay (Supplementary Figure 2). The results showed that KCBE ( ≤ 200 μg/ml), QMG ( ≤ 20 μM), and CG ( ≤ 20 μM) maintained cell viability above 80%. Accordingly, the highest non-cytotoxic concentrations were selected for subsequent experiments.

SA-β-gal staining (Figure 4A) showed that MGO induced a typical senescent phenotype in Fbs, characterized by a significant increase in SA-β-gal-positive cells, enlarged cell size, and a flattened morphology. Treatment with KCBE, QMG, or CG significantly reduced senescent cell proportions (Figure 4B), with QMG showing the strongest effect, reducing senescence by 74.1% (*P* < 0.001).

**Figure 4:**
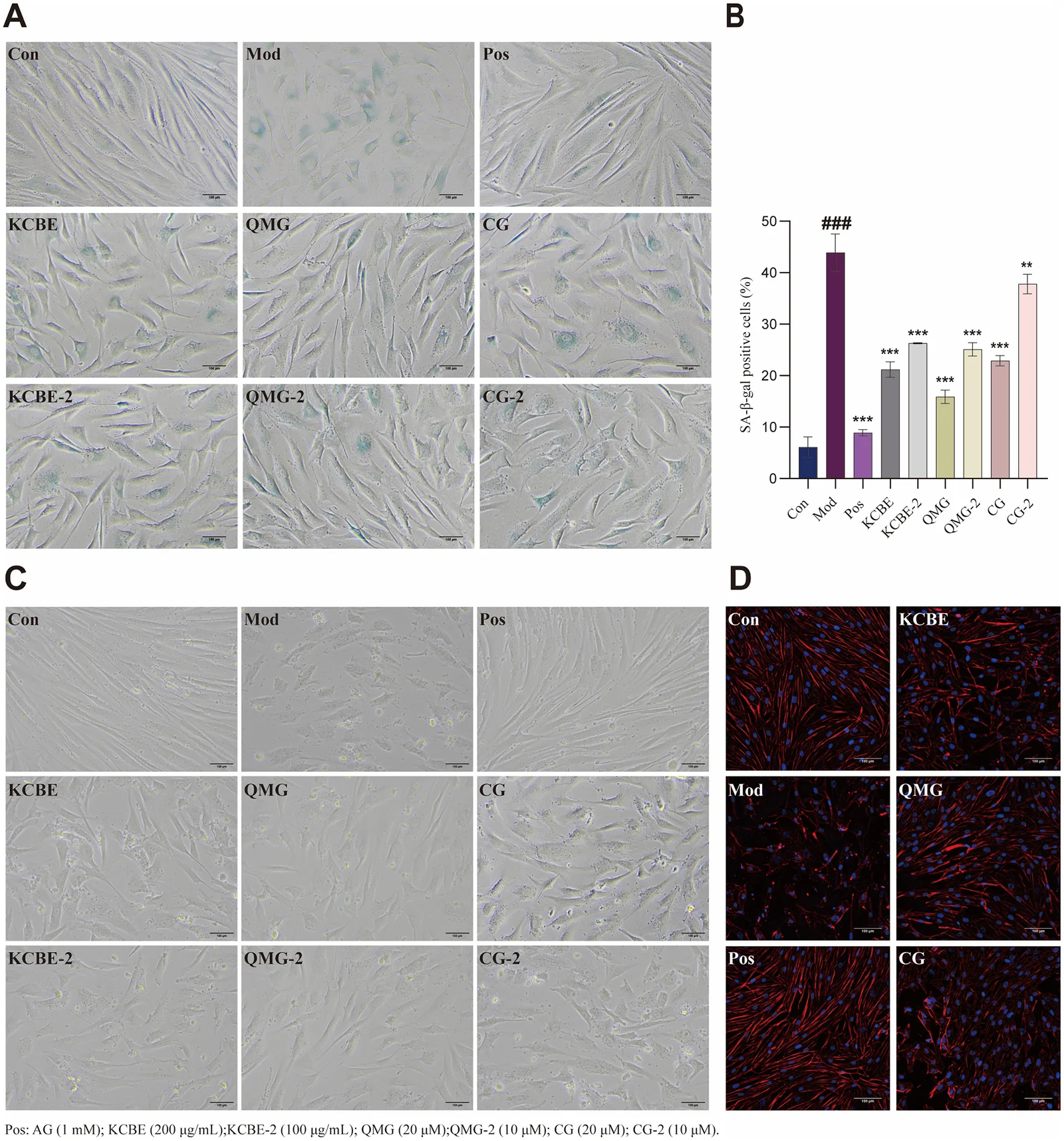
Protective effects of KCBE, QMG and CG against MGO-induced senescence in human skin-derived fibroblasts. **(A)** Representative light microscopy images of senescent cells stained with SA-β-gal (scale bar = 100 μm). **(B)** Quantitative analysis of the percentage of SA-β-gal-positive cells. **(C)** Representative phase-contrast images showing cell morphological changes (scale bar = 100 μm). **(D)** Representative fluorescence images of F-actin cytoskeleton staining (red) counterstained with DAPI for nuclei (blue) (scale bar = 100 μm). Data are expressed as the mean ± SD (*n* = 3). ###*P* < 0.001 for the Mod group compared with the Con group; **P < 0.01, ***P < 0.001 for the treatment groups vs. the Mod group.

Morphological observations (Figure 4C) further confirmed that MGO-induced hypertrophic phenotypes were reversed after treatment, with cells regaining a typical spindle-shaped fibroblast morphology at 72 h, consistent with the morphological improvements observed at 24 and 48 h (Supplementary Figure 3). Immunofluorescence analysis (Figure 4D) demonstrated that MGO caused severe disruption of the F-actin cytoskeleton, whereas treatment restored filament organization, with actin fibers realigning along the longitudinal axis, indicating recovery of cytoskeletal integrity.

### 3.5 Effects of KCBE, QMG, and CG on collagen I deposition and intracellular ROS levels

Immunofluorescence analysis (Figure 5A) showed that, compared with the model group, treatment with KCBE, QMG, and CG significantly increased the mean fluorescence intensity of COL I (Figure 5B) by 91.0%, 94.8%, and 95.0%, respectively (all *P* < 0.001), indicating that KCBE, QMG and CG effectively attenuated glycation-induced loss of extracellular COL I matrix.

**Figure 5:**
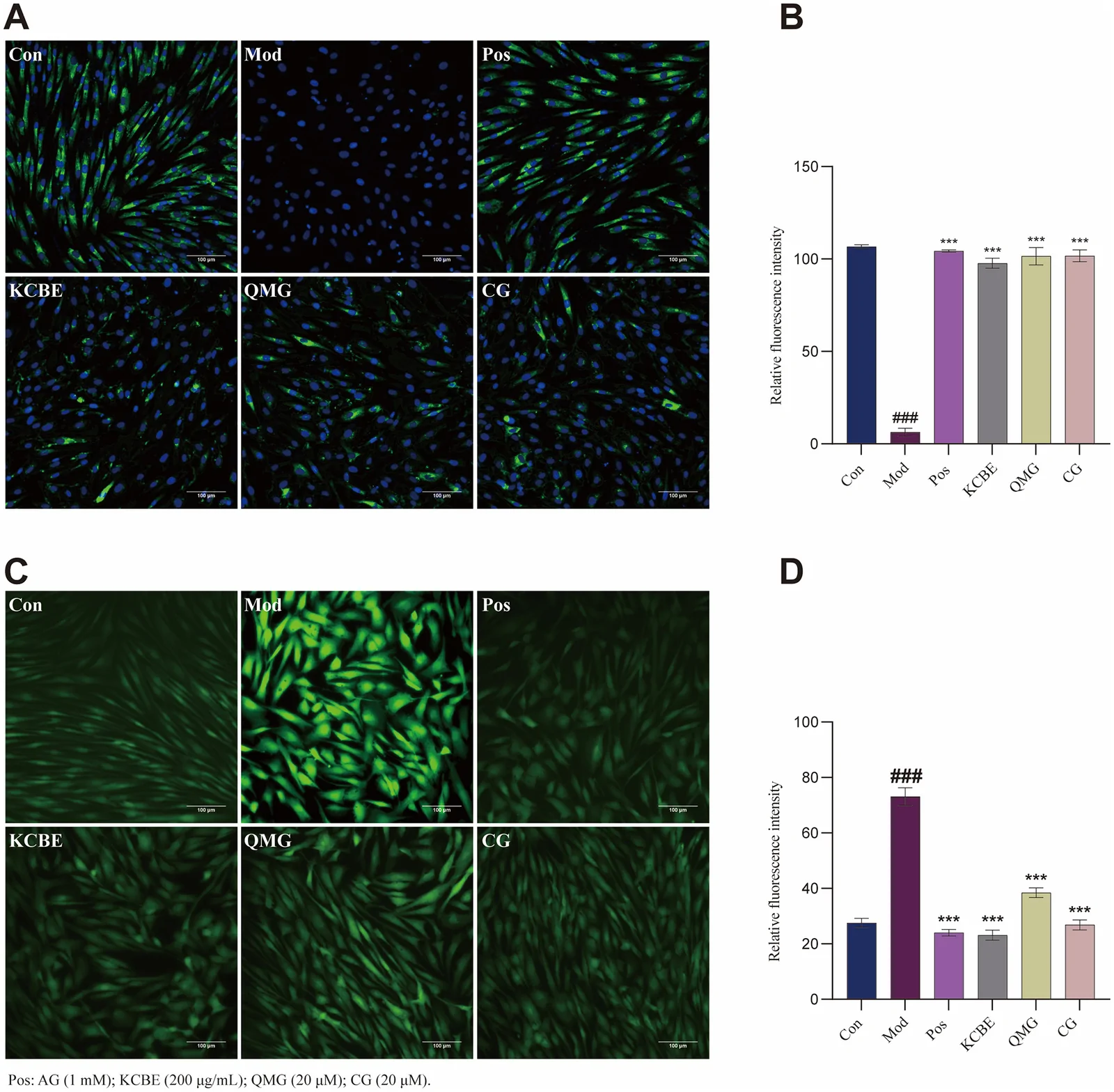
Regulatory effects of KCBE, QMG and CG on COL I deposition and intracellular ROS production. **(A)** Representative immunofluorescence images of COL I expression (green) with DAPI counterstaining for nuclei (blue) (scale bar = 100 μm). **(B)** Quantitative analysis of relative fluorescence intensity. **(C)** Representative fluorescence images of intracellular ROS accumulation (green) (scale bar = 100 μm). **(D)** Quantitative analysis of relative ROS fluorescence intensity. Data are expressed as the mean ± SD (*n* = 3). ###*P* < 0.001 for the Mod group compared with the Con group; ***P < 0.001 for the treatment groups vs. the Mod group.

ROS fluorescence analysis (Figure 5C) showed that intracellular ROS levels (Figure 5D) were significantly reduced following treatment with KCBE, QMG, and CG, showing decreases of 76.0%, 101.6%, and 109.7%, respectively, compared with the model group (all *P* < 0.001). Notably, ROS levels in both monomer-treated groups were reduced to below those of the blank control group. These findings indicate that KCBE, QMG and CG effectively scavenge excessive reactive oxygen species and alleviate glycation-induced oxidative stress in Fbs.

### 3.6 Inhibitory effects of KCBE, QMG, and CG on MGO-induced inflammatory responses

In the MGO-induced senescence model, AGEs can promote the release of pro-inflammatory cytokines via the RAGE/NF-κB signaling pathway, thereby exacerbating glycation-induced aging. ELISA results showed that MGO induction significantly elevated the secretion levels of IL-1β, IL-6, and IL-8 (Figures 6A–C) in the cell supernatant compared with the blank control group (all *P* < 0.001). Following KCBE intervention, the secretion levels of these three pro-inflammatory cytokines were markedly reduced by 112.0%, 135.9%, and 132.1%, respectively, compared with the model group (all *P* < 0.001), returning to below normal levels. Consistently, both QMG and CG treatments exhibited similar inhibitory effects. These findings indicate that KCBE, QMG and CG can effectively suppress MGO-induced pro-inflammatory cytokine release.

**Figure 6:**
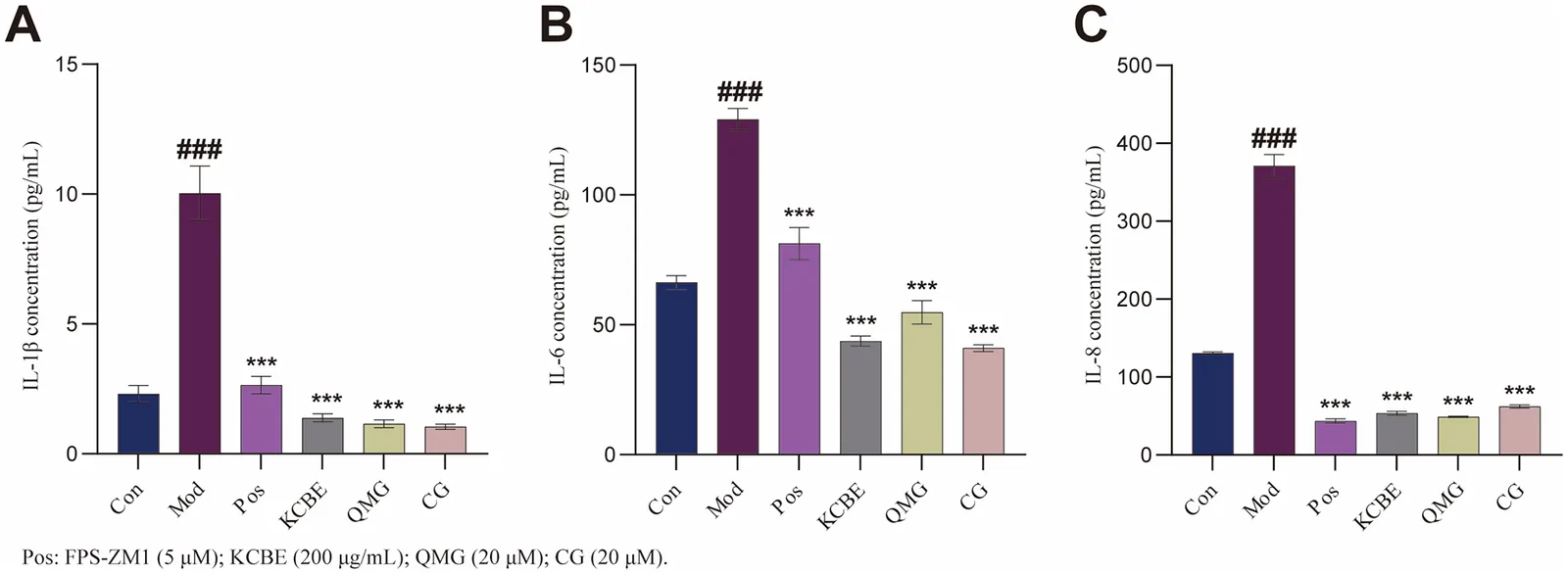
Inhibitory effects of KCBE, QMG and CG on MGO-induced inflammatory cytokine secretion. The secretion levels of pro-inflammatory cytokines, including **(A)** IL-1β, **(B)** IL-6, and **(C)** IL-8 in the cell culture supernatants, were determined by ELISA. Data are presented as the mean ± SD (*n* = 3). ###*P* < 0.001 for the Mod group compared with the Con group; ***P < 0.001 for the treatment groups vs. the Mod group.

### 3.7 Regulation of ECM-related gene expression by KCBE, QMG, and CG

#### 3.7.1 Inhibition of the RAGE-mediated NF-κB (p65) signaling pathway

qPCR analysis showed that, compared with the model group, KCBE treatment significantly downregulated the mRNA expression levels of RAGE, NF-κB, and p65 (Figures 7A–C) by 47.1%, 89.4%, and 77.9%, respectively (*P* < 0.001, *P* < 0.001, *P* < 0.05). Both QMG and CG exhibited even more pronounced inhibitory effects; specifically, QMG reduced RAGE, NF-κB, and p65 expression by 93.0%, 109.2%, and 114.6%, respectively, while CG suppressed them by 88.6%, 103.8%, and 115.3%, respectively (all *P* < 0.001). Notably, the transcription levels of NF-κB and p65 in both monomer-treated groups were restored to near or below control levels. These results demonstrate that KCBE, QMG and CG strongly inhibit the activation of the RAGE/NF-κB (p65) signaling pathway at the transcriptional level, with QMG and CG showing superior efficacy to the crude extract.

**Figure 7:**
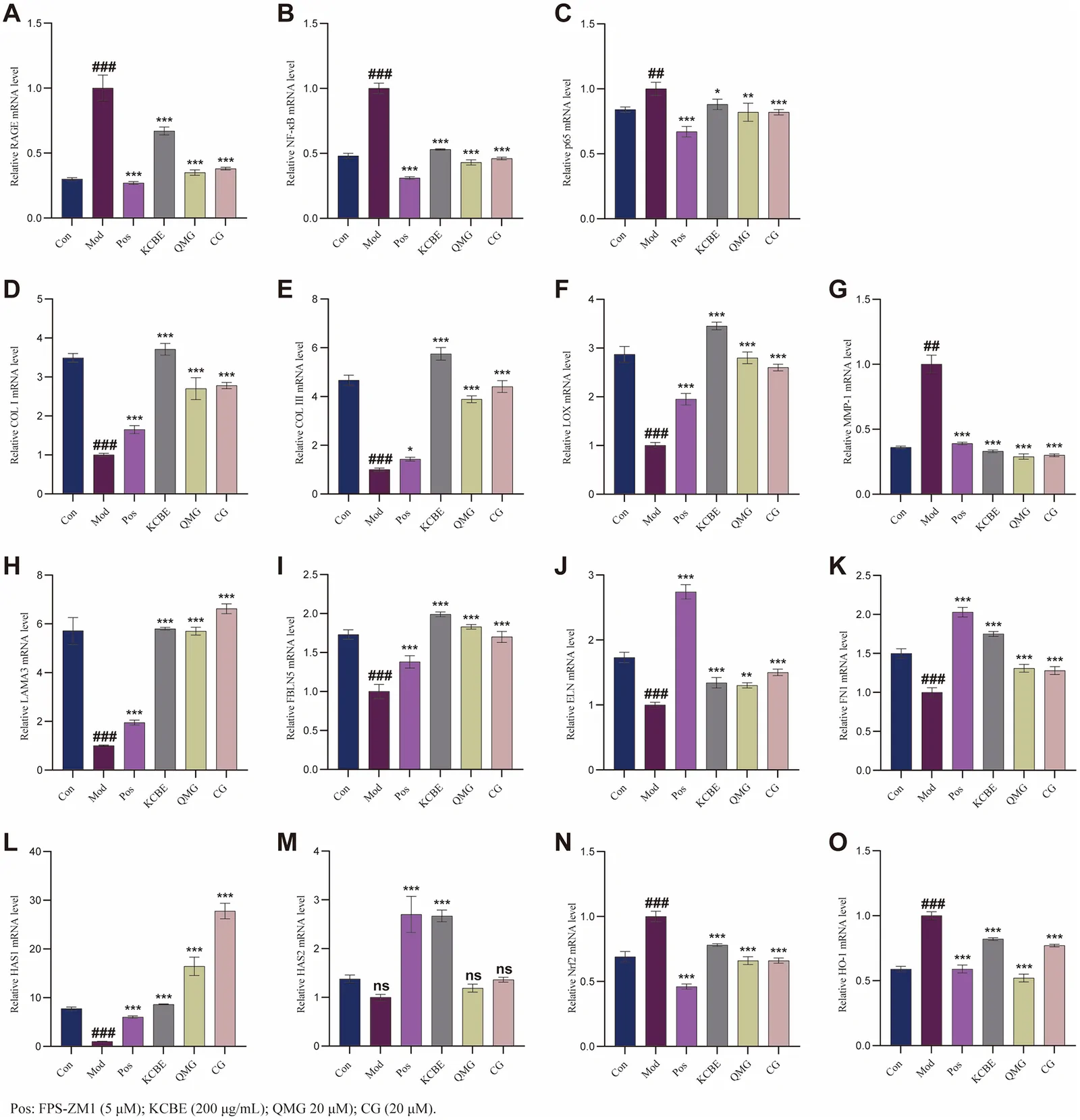
Effects of KCBE, QMG, and CG on the mRNA expression levels of genes related to inflammation, extracellular matrix (ECM), and oxidative stress. The relative mRNA expression levels of **(A)** RAGE, **(B)** NF-κB, **(C)** p65, **(D)** COL I, **(E)** COL III, **(F)** LOX, **(G)** MMP-1, **(H)** LAMA3, **(I)** FBLN5, **(J)** ELN, **(K)** FN1, **(L)** HAS1, **(M)** HAS2, **(N)** Nrf2, and **(O)** HO-1 were determined by qPCR. Data are presented as mean ± SD (*n* = 3). ##*P* < 0.01, ###*P* < 0.001 for the Mod group compared with the Con group; *P < 0.05, **P < 0.01, ***P < 0.001 for the treatment groups vs. the Mod group; ns, non-significant.

#### 3.7.2 Amelioration of ECM remodeling-related genes

The balance between collagen synthesis and degradation is central to maintaining skin ECM homeostasis. qPCR results showed that, compared with the model group, KCBE treatment significantly upregulated the mRNA expression levels of collagen I (COL I), collagen III (COL III), and lysyl oxidase (LOX) (Figures 7D–F) by 108.8%, 129.4%, and 131.4%, respectively (all *P* < 0.001), while downregulating the expression of the collagen degradation enzyme MMP-1 (Figure 7G) by 104.2% (*P* < 0.001). Both QMG and CG treatments exhibited a consistent regulatory trend, showing varying degrees of upregulation for COL I, COL III, and LOX, while significantly suppressing MMP-1 expression by 110.7% and 109.4%, respectively (all *P* < 0.001). The concurrent upregulation of COL I/COL III together with LOX, accompanied by MMP-1 suppression, indicates enhanced collagen biosynthesis and maturation while reducing collagen degradation, thereby favoring collagen deposition within the ECM. These findings demonstrate that KCBE can simultaneously promote collagen synthesis and cross-linking while inhibiting collagen degradation, with QMG and CG playing a vital role in these processes.

#### 3.7.3 Maintenance of basement membrane stability genes

Laminin subunit alpha 3 (LAMA3) and fibulin 5 (FBLN5) are crucial genes for maintaining the structural integrity of the epidermal-dermal basement membrane. qPCR results showed that, compared with the model group, KCBE treatment significantly upregulated the mRNA expression levels of LAMA3 and FBLN5 (Figures 7H, I) by 101.7% and 136.1%, respectively (all *P* < 0.001). Furthermore, QMG treatment upregulated their expression by 99.7% and 113.2%, respectively (all *P* < 0.001), while CG treatment increased them by 119.1% and 96.3%, respectively (all *P* < 0.001). Notably, CG exhibited the most prominent upregulating effect on LAMA3, whereas KCBE showed the greatest overall enhancement of FBLN5 expression. These findings suggest that QMG and CG may exert a synergistic effect in maintaining basement membrane stability.

#### 3.7.4 Enhancement of ECM structural and adhesion genes

Elastin (ELN) and fibronectin 1 (FN1) are involved in elastic fiber formation, matrix adhesion, and structural assembly, respectively. qPCR results showed that, compared with the model group, KCBE treatment significantly upregulated the mRNA expression levels of ELN and FN1 (Figures 7J, K) by 46.6% and 49.5%, respectively (all *P* < 0.001). Furthermore, QMG treatment upregulated their expression by 41.5% and 61.9%, respectively (*P* < 0.01, *P* < 0.001), while CG treatment increased them by 68.1% and 55.7%, respectively (all *P* < 0.001). Notably, CG exhibited a more prominent upregulating effect on ELN, whereas KCBE showed a higher enhancement of FN1 expression than either QMG or CG administered alone. These findings indicate that KCBE, QMG and CG play an essential role in maintaining ECM structural stability.

#### 3.7.5 Promotion of hyaluronic acid synthesis-related genes

Hyaluronan synthase 1 (HAS1) and hyaluronan synthase 2 (HAS2) are key enzyme-coding genes involved in hyaluronic acid synthesis. qPCR results showed that, compared with the model group, KCBE treatment significantly upregulated the mRNA expression levels of HAS1 and HAS2 (Figures 7L, M) by 113.0% and 439.9%, respectively. Both QMG and CG exhibited a more pronounced upregulating effect on HAS1, with QMG and CG increasing its expression by 228.8% and 396.8%, respectively (all *P* < 0.001). Conversely, regarding HAS2 expression, the upregulating effect of KCBE (439.9%) was superior to that of either QMG or CG administered alone, further demonstrating that the multi-component profile of KCBE exerts a synergistic regulation on hyaluronic acid synthesis.

#### 3.7.6 Modulation of antioxidant response-related genes

Nuclear factor erythroid 2–related factor 2 (Nrf2) acts as the master transcription factor for antioxidant defense, while heme oxygenase-1 (HO-1) is a pivotal downstream phase II enzyme responsible for scavenging free radicals. qPCR results showed that, compared with the model group, KCBE treatment downregulated the mRNA expression levels of Nrf2 and HO-1 (Figures 7N, O) by 73.1% and 45.4%, respectively (all *P* < 0.001). Both QMG and CG exhibited even more pronounced downregulating effects; specifically, QMG treatment reduced Nrf2 and HO-1 expression by 110.1% and 117.0%, respectively (all *P* < 0.001), while CG treatment decreased them by 109.9% and 55.4%, respectively (all *P* < 0.001). This downregulation of Nrf2/HO-1 expression likely reflects a feedback normalization of the cellular antioxidant system after MGO-induced excessive oxidative stress was effectively eliminated by the treatments.

## 4 Discussion

Skin glycation-associated aging is considered one of the key manifestations of organismal aging, primarily driven by the progressive accumulation of AGEs, which impair dermal microenvironment homeostasis and cellular functions (24). AGEs not only alter extracellular matrix (ECM) architecture but also activate receptor for AGEs (RAGE)-mediated signaling pathways, triggering downstream cascades of oxidative stress and inflammatory responses, ultimately accelerating structural and functional deterioration of the skin (11).

UPLC-QTOF-MS profiling revealed that KCB is primarily composed of flavonoid glycosides and phenolic acids. Specifically, the characterization identified one organic acid (compound **1**), five flavonoid glycosides (compounds **9**, **10**, **11**, **12**, and **13**), and seven phenolic acids (compounds **2**, **3**, **4**, **5**, **6**, **7**, and **8**). The life cycle of KCB transitions through four distinct flowering stages (KCB1–KCB4). Dynamic UPLC-MS/MS quantification across these periods demonstrated that the cumulative content of the six major target compounds (**3**, **4**, **7**, **9**, **10**, and **11**) in KCB3 extracts was significantly higher than that in any other growth stage. Among these, compound **3** (CG) was established as the predominant phenolic acid constituent within the plant matrix. Enzyme inhibition assays further revealed that CG exhibited potent antioxidant activity, together with marked tyrosinase and collagenase inhibitory activities, highlighting it as a key bioactive constituent responsible for the antioxidant, skin-whitening, and anti-collagen degradation properties of the extract. Although rutin (compound **9**) exhibited the highest intrinsic capacity in both antioxidant and anti-glycation assays, its abundance in KCB was exceedingly restricted (<0.3%). compound **11**(QMG) maintained a persistent concentration approximately 5- to 8-fold greater than that of rutin across all flowering stages, while demonstrating robust antioxidant and anti-glycation performance. Given their relatively high abundance and confirmed bioactivities, CG and QMG were selected as representative phenolic acid and flavonoid constituents for subsequent cellular validation.

Cellular senescence is characterized by functional decline and cytoskeletal disorganization. Glycation stress induces abnormal crosslinking of both extracellular and intracellular structural proteins, such as vimentin and tubulin, thereby disrupting cytoskeleton dynamics and accelerating senescence progression (25, 26). In this study, MGO-induced Fbs exhibited typical senescent phenotypes, including increased SA-β-gal positivity and pronounced cytoskeletal disruption. Treatment with KCBE effectively alleviated these changes. These findings suggest that KCBE may help preserve cytoskeletal integrity under glycation-associated stress. Concurrently, this intracellular F-actin restoration was parallel to the enhanced deposition of extracellular type I collagen, which may contribute to the preservation of dermal ECM homeostasis.

Oxidative stress is a major amplification mechanism in AGEs-induced skin aging. AGEs activate RAGE-mediated NADPH oxidase signaling, leading to excessive ROS production and persistent oxidative damage (27, 28). In parallel, AGEs also activate NF-κB signaling, promoting the expression of pro-inflammatory cytokines such as IL-1β and IL-6, thereby exacerbating inflammatory injury (29). In this study, KCBE treatment significantly reduced intracellular ROS levels and suppressed IL-1β, IL-6, and IL-8 secretion, indicating effective attenuation of oxidative stress and inflammatory responses. Notably, downregulation of NF-κB/p65 suggests suppression of inflammatory transcriptional activity. In addition, MGO exposure induced upregulation of Nrf2 and HO-1, while treatment partially normalized their expression. The Nrf2/HO-1 axis is a major component of the cellular antioxidant defense system, and increased Nrf2 and HO-1 mRNA expression may represent a compensatory cellular response to excessive oxidative stress (30). Consistent with this interpretation, a previous study reported that oxidative stress induced by sulfamethoxazole was accompanied by compensatory upregulation of Nrf2 and its downstream antioxidant genes, including HO-1, whereas lycopene treatment alleviated oxidative stress and restored their expression toward basal levels (31). Based on these findings, we speculate that the reduction in Nrf2 and HO-1 mRNA levels following KCBE treatment primarily reflects normalization of the compensatory antioxidant response as oxidative stress is alleviated. However, the possibility that KCBE may also exert a direct regulatory effect on the Nrf2/HO-1 pathway cannot be completely ruled out.

Continuous AGE accumulation further aggravates glycation-associated damage and disrupts ECM homeostasis through aberrant activation of matrix-degrading factors such as MMPs, ultimately contributing to collagen degradation, loss of elasticity, and structural deterioration of the skin (1). In the present study, MGO-induced senescence was accompanied by widespread dysregulation of ECM-related genes. Elevated MMP-1 expression indicated enhanced matrix degradation, whereas reduced COL I and COL III expression suggested impaired collagen biosynthesis. The downregulation of ELN and FN1 reflected suppression of elastic fiber formation and ECM scaffold assembly. In addition, decreased LOX expression implied impaired collagen and elastin crosslinking, which may further compromise ECM structural stability. Reduced expression of LAMA3 and FBLN5 suggested disruption of basement membrane integrity and epidermal-dermal adhesion. Furthermore, dysregulation of HAS1 and HAS2 may affect hyaluronic acid homeostasis, thereby weakening skin hydration and elasticity maintenance. However, treatment with KCBE effectively reversed these alterations in ECM-related gene expression, indicating its potential to preserve ECM homeostasis and mitigate glycation-induced dermal damage. Interestingly, although QMG exhibited no appreciable direct collagenase inhibitory activity in the *in vitro* collagenase inhibition assay, it markedly suppressed MMP-1 mRNA expression and promoted COL I deposition in MGO-induced Fbs. Since collagen homeostasis is maintained by the dynamic balance between collagen synthesis and degradation, enhanced collagen synthesis together with reduced MMP-1 expression can still favor net collagen accumulation in the absence of direct collagenase inhibition. Thus, QMG may promote collagen accumulation primarily through transcriptional regulation of MMP-1 and collagen biosynthesis rather than direct enzyme inhibition.

This study also has some limitations that should be acknowledged. Although the MGO-induced skin fibroblast model is a well-established *in vitro* approach for investigating glycation-associated skin damage (including skin aging) and has been validated in several related studies (29, 32–34), it does not fully recapitulate the chronic nature of AGE accumulation in human skin. In the dermis, AGEs gradually accumulate over time under the combined influence of aging, dietary factors, and environmental exposures, representing a chronic and progressive process. In contrast, our model induces glycation-associated damage through short-term exposure to a relatively high concentration of MGO, which rapidly produces acute cellular stress and cannot fully reproduce the gradual, low-level accumulation of AGEs that occurs in human skin. Moreover, a more systematic evaluation of the anti-aging effects of KCBE in other skin-relevant cell types, particularly keratinocytes and immune cells, is warranted to better understand its potential effects across different cellular components of the skin. Further validation using more physiologically relevant models, such as skin organoids or reconstructed skin models, would also help to clarify the protective effects of KCBE within a more integrated skin microenvironment.

In summary, KCB exerts anti-glycation and anti-skin aging effects through the synergistic actions of phenolic acids and flavonoids, targeting multiple pathways including AGE formation, oxidative stress, inflammatory signaling, and ECM homeostasis. This study provides mechanistic evidence supporting its potential as a natural anti-aging functional ingredient. Given the abundant accumulation of bioactive compounds at the KCB3 stage, these findings may support the further evaluation of KCB for anti-skin-aging applications.

## 5 Conclusion

In this study, UPLC-QTOF-MS and UPLC-MS/MS technologies were applied to perform a systematic qualitative and quantitative analysis of the chemical constituents of KCB. A total of 13 major compounds were identified and categorized into organic acids, flavonoids, and phenolic acids. Phytochemical profiling revealed that the half-bloom stage (KCB3) exhibited the highest cumulative content of six core compounds, establishing it as the optimal harvest window. Among these, the representative phenolic acid, CG (compound **3**), accounted for over 60% of the total target constituents, while QMG (compound **11**) was identified as the predominant flavonoid monomer.

Enzymatic and Antioxidant assays demonstrated that the phenolic acids (compounds **3**, **4**, and **7**) possessed versatile antioxidant, anti-tyrosinase, and anti-collagenase activities. Conversely, the flavonoids (compounds **9**, **10**, and **11**) predominantly contributed to antioxidant and anti-glycation capacities. These two compound classes conferred multi-functional bioactivities to the crude extract. Furthermore, KCBE and its characteristic monomers (QMG and CG) were selected for *in vitro* cellular validation using an MGO-induced Fbs senescence model. Their anti-aging efficacy was comprehensively evaluated across multiple dimensions, including senescent phenotypes, cytoskeletal networks, oxidative stress, inflammatory microenvironments, and ECM metabolism. The results demonstrated that KCBE and both monomers reduced the proportion of senescent cells, improved cytoskeletal organization, suppressed pro-inflammatory cytokine secretion, and increased type I collagen deposition, accompanied by changes in the expression of ECM-related genes. Overall, the findings indicate that KCBE and its representative compounds exert protective effects against MGO-induced cellular damage and provide a basis for further investigation of KCB bioactive compounds for anti-skin-aging applications.

## Data Availability

The original contributions presented in this study are included in the article and Supplementary material. Further inquiries can be directed to the corresponding authors.
