# Supplementary material for: Kanzan cherry blossom and its active compounds attenuate methylglyoxal-induced skin fibroblast senescence through RAGE/NF-κB suppression and ECM restoration
Source: Front Nutr. 2026 Oct 28;13:1927058. doi: 10.3389/fnut.2026.1927058 (PMC13751434; doi:10.3389/fnut.2026.1927058)
Supplement: Supplementary file 1 [file Table_1.docx]

Supplementary Material

# 1 Supplementary Table

**Supplementary Table 1.** Primer sequences used for qPCR analysis.

| **Primer name** | **Primer sequence（5’→3’）** |
| --- | --- |
| GADPH | FP: GGAGCGAGATCCCTCCAAAAT  RP: GGCTGTTGTCATACTTCTCATGG |
| RAGE | FP: AGCCACTGGTGCTGAAGTGTAAGG  RP: AGAGGGAGCCGTTGGGAAGGAC |
| NF-κB | FP: GGATTTCGTTTCCGTTATGT  RP: GTCCTTGGGTCCAGCAGTTA |
| p65 | FP: GGGGACTACGACCTGAATGCT  RP: GGGCACGATTGTCAAAGATGG |
| Nrf2 | FP: ACACGGTCCACAGCTCATC  RP: TGTCAATCAAATCCATGTCCTG |
| HO-1 | FP: GCGGGCCAGCAACAAAGTGCAAGA  RP: AAGGACCCATCGGAGAAGCGGAGC |
| COL I | FP: GTTTCGACTTCAGCTTCCTGC  RP: GGTTCTTGCGGCTTCCCTCT |
| COL III | FP: CTTGGTCAGTCCTATGCGGAT  RP: GGGTTGGGGCAGTCTAATTC |
| MMP-1 | FP: GGGCTGAAAGTGACTGGGAAAC  RP: CTTGGCAAATCTGGCGTGTAAT |
| ELN | FP: GGTGGCTTAGGAGTGTCTG  RP: GGCAGTTTCCCTGTGGTGTAG |
| FN1 | FP: GCTCAGCAAATCGTGCAGC  RP: CTAGGTAGGTCCGTTCCCACT |
| LAMA3 | FP: TCAATGCCAGGATGGCTCAG  RP: TATCCCGGTGCACACCTTTC |
| FBLN5 | FP: ATGTCGCTATGGTTACTGCC  RP: GAAAGAGCCGTAGGTGTTGA |
| LOX | FP: GATGCCAACACCCAGAGGAGAG  RP: GGTATCATAACAGCCAGGACTC |
| HAS1 | FP: AACCTCTTGCAGCAGTTTCTTG  RP: CGACTTGGACCAGCGTGTCT |
| HAS2 | FP: TCATCATCCAAAGCCTGTT  RP: ATCTTCTGAGTTCCCATCTA |

# 2 Supplementary Figures

**Supplementary Figure 1.** Supplier‑provided documentary evidence for the *Cerasus serrulata* 'Sekiyama' (Kanzan cherry) raw material. Scanned copy of an official statement issued by Taian Cherry Blossom Seedling Planting and Sales Professional Cooperative. The document describes the cultivation history, phenotypic traits, planting acreage and annual fresh flower output of Guanshan cherry at the planting base, to authenticate the cultivar origin of the KCBE starting material.

**Supplementary** **Figure 2.** Effects of KCBE and its active monomers on cell viability.Cell viability of Fbs was evaluated by CCK-8 assay after treatment with various concentrations of (A) KCBE (0–10000 μg/mL), (B) QMG (0–200 μM), and (C) CG (0–200 μM) Data are expressed as the mean ± SD (n = 3).

**Supplementary Figure 3.** Time-dependent protective effects of KCBE, QMG, and CG on MGO-induced morphological changes in Fbs. Representative phase-contrast micrographs showing the morphology of Fbs after MGO induction and treatment for 24 h and 48 h.
